# A *Fusarium* Isolate from a Salt Marsh Improves the Salinity Tolerance of a Commercial Cultivar of *Festuca rubra* via Enhanced Root K^+^ Homeostasis

**DOI:** 10.3390/microorganisms14071598

**Published:** 2026-07-22

**Authors:** Liping Wang, Sasirekha Munikumar, Junjie Yi, Marten Staal, Jan Henk Venema, Theo Elzenga

**Affiliations:** 1 Plant Ecophysiology Group, Groningen Institute for Evolutionary Life Sciences, University of Groningen, 9747 AG Groningen, The Netherlands; m.staal@rug.nl (M.S.); j.h.venema@rug.nl (J.H.V.); 2 Research Center for Ecological Remediation of Mining & Metallurgical Sites, Central South University, Changsha 410083, China; 3 Biostimulants (R&D), Growing Solutions, ICL, Nijverheidsweg 1, 6422 PD Heerlen, The Netherlands; sasirekha.munikumar@icl-group.com; 4 Integrated Research on Energy, Environment and Society (IREES), University of Groningen, 9747 AG Groningen, The Netherlands; yijunjie06000@126.com

**Keywords:** potassium flux, salinity stress, plant microbiome interactions, fungal isolate, *Festuca rubra* ssp. *rubra*, *Fusarium*

## Abstract

Salinity poses a major threat to sustainable agriculture and coastal ecosystems, resulting in a substantial loss of plant productivity and biodiversity. Although some coastal grass species exhibit natural adaptation to saline conditions, the physiological mechanisms underlying salt tolerance remain incompletely understood, particularly regarding the contribution of plant-associated microorganisms. In a previous study, a commercial cultivar of red fescue (*Festuca rubra* ssp. *rubra* cv. Rafael) was shown to be salt sensitive when grown hydroponically, whereas wild populations of *F. rubra* commonly occur in coastal salt marshes (possibly ssp. *litoralis*). We hypothesized that this difference in salt tolerance is partly associated with beneficial fungal plant interactions. To test this hypothesis, we investigated whether inoculation with a fungal isolate designated *Fusarium* sp. 1 and isolated from *F. rubra* growing on a salt marsh along the Dutch Wadden Sea coast could improve the salinity tolerance of the commercial cultivar. The results showed that inoculation with *Fusarium* sp. 1 alleviated the salt-induced growth inhibition. At 100 mM NaCl, shoot and root biomass were partially restored relative to non-inoculated controls, accompanied by a significant increase in the shoot-to-root ratio. To investigate the physiological basis of this response, we applied the Microelectrode Ion Flux Estimation (MIFE) technique to quantify Na^+^ -induced K^+^ efflux in roots. Inoculated plants exhibited improved K^+^ homeostasis, characterized by a reduced instantaneous Na^+^-induced K^+^ efflux and a faster recovery of root fluxes. Moreover, inoculated plants grown at 50 and 100 mM NaCl displayed 333% and 397% greater net K^+^ influx, respectively, compared with non-inoculated controls. Our results indicated that inoculation with *Fusarium* sp. 1 improves the salinity tolerance of *F. rubra,* likely through enhanced root K^+^ retention. These findings suggest that commercial *F. rubra* cultivars remain responsive to beneficial microbial associations and highlight the potential of exploring plant–microbe interactions from naturally salt-adapted environments to improve salinity resilience in grasses and potentially other crops.

## 1. Introduction

The increase in soil salinization has resulted in a dramatic global loss of agricultural land, estimated at nearly one billion hectares, and has reduced crop productivity on about 20% of irrigated land worldwide [1,2,3,4]. This salinization poses a growing challenge for agriculture to meet the global demand for food, fodder and bioenergy, while also threatening ecosystem diversity. The adverse effects of salinity on plants include, but are not limited to, osmotic stress, Na^+^ and Cl^−^ toxicity, and subsequent oxidative stress. Some plant species cope with salinity by excluding salt to prevent ion toxicity or by limiting salt entry to maintain osmotic balance through multiple physiological processes [5,6,7,8]. However, most crop species are salt-sensitive and lack this acclimation capacity.

Roles for microorganisms in mitigating stress effects in plants have been widely reported over the last few decades [7,8,9,10,11,12,13]. Numerous rhizosphere and plant-associated bacterial and fungal species have been identified as capable of promoting plant growth, enhancing tolerance to abiotic stress and providing protection against pathogens [14,15,16]. Inoculation with fungal isolates has been shown to improve growth and stress tolerance in several plant species, including rice (*Oryza sativa*), barley (*Hordeum vulgare*), maize (*Zea mays*), tomato (*Solanum lycopersicum*), soybean (*Glycine max*), alfalfa (*Medicago truncatula*) and *Arabidopsis thaliana* [16,17,18,19,20,21,22,23,24,25,26,27,28]. Interactions between root-associated fungal isolates and grasses have also been shown to enhance host plant growth, biomass and quality [16,29]. However, there is increasing concern that traditional breeding and modern cultivation practices may inadvertently reduce plant microbiome associations, potentially limiting the contribution of microorganisms to stress tolerance [30,31].

Fungal isolates associated with plant roots in extreme environments are hypothesized to possess adaptive traits that enhance the stress tolerance of their host plants, often in a habitat-specific manner [13,14,32,33,34]. Many of these fungi produce phytohormones that are also synthesized by plants and play important roles in regulating growth and stress responses [22,35,36,37]. Native plants from saline environments frequently exhibit high salinity tolerance, a trait that may be closely linked to their associations with root-associated microbes [14,38,39,40]. Certain fungal isolates isolated from halophytic plant species have been demonstrated to be tolerant to high NaCl concentrations in vitro [41]. Moreover, inoculation of salt-sensitive wheat genotypes with fungal isolates from halophytic plants improved seed germination and enhanced growth under saline conditions [16,42]. Similarly, fungal isolates from native halophytic *Hordeum secalinum* increased yield in commercial barley varieties, highlighting their potential agricultural relevance [43,44,45] reported that soybean seeds primed with the fungal isolate *Fusarium moniliforme* showed improved germination and growth under salt stress. *Fusarium* isolates have also been reported to suppress pathogenic fungi and enhance resistance against *Botrytis cinerea* [28]. Grasses are particularly common in saline habitats, yet fungal–root associations in grasses remain comparatively underexplored.

The mechanisms by which these fungi improve stress tolerance are not yet fully understood. High-affinity K^+^ transporters (HKTs) are found in plants, bacteria and fungi and belong to a large transporter family. Evidence suggests that such fungal associations do not induce entirely novel stress-tolerance mechanisms, but rather activate or enhance inherent plant acclimation strategies, including antioxidant defense systems, phytohormone production and eco-physiological processes such as water relations, gas exchange and ion homeostasis mediated by transporter families such as HKTs and NHXs [17,18,19,24,44,46].

K^+^ efflux has been shown to be a reliable indicator of salt tolerance and can therefore be used to assess inherent salinity tolerance in plants, which may be further enhanced by associations with endophytic microbiomes [4,47,48,49,50]. The instantaneous change in K^+^ efflux upon NaCl exposure and the subsequent recovery to a new steady state are considered indicative of plant salinity tolerance [44,51,52]. Comparative studies in barley, wheat, grapevine rootstock and iris demonstrated a strong relationship between K^+^ retention capacity and salt tolerance [53,54,55,56]. In barley, K^+^ efflux measured in the mature root zone of seven cultivars showed a very strong negative correlation with shoot dry weight (r^2^ = 0.96), plant height (r^2^ = 0.94) and flag leaf osmolarity (r^2^ = 0.91) [53]. Salt-tolerant cultivars exhibited substantially lower K^+^ efflux (20–25 nmol·m^−2^·s^−1^) than salt-sensitive cultivars (150–180 nmol·m^−2^·s^−1^). These findings highlight the importance of Na^+^ exclusion, maintenance of an optimal K^+^: Na^+^ ratio, and K^+^ retention as key determinants of plant salinity tolerance.

In this study, we tested a fungal isolate, designated *Fusarium* sp. 1, previously isolated from salt marsh-grown red fescue (*Festuca rubra*), for its potential to enhance salinity resilience in a commercial red fescue cultivar (*F. rubra* ssp. *rubra* cv. Rafael). According to [57], the salt-marsh (SM) ecotype *F. rubra* most likely corresponds to ssp. *litoralis*. Previous studies showed that the commercial cultivar *F. rubra* ssp. *rubra* cv. Rafael is salt-sensitive [58,59], whereas wild *F. rubra* populations inhabit the Dutch coast at high salinity (around 12 ppt, ≈205 mM NaCl). This study therefore focuses on the physiological consequences of fungal isolates from naturally salt-adapted populations and on whether salinity tolerance could be partially restored in the commercial cultivar. Inoculated and non-inoculated plants were grown under different NaCl concentrations. The Microelectrode Ion Flux Estimation (MIFE) technique was employed to quantify K^+^ fluxes in response to salt stress. This study aimed to address the following questions: Can inoculation with *Fusarium* sp. 1, isolated from an *F. rubra* ecotype growing in a Dutch salt marsh, improve the growth and salinity tolerance of the salt-sensitive commercial cultivar *F. rubra* ssp. *rubra* cv. Rafael? If so, is the improvement in salinity tolerance related to enhanced K^+^ homeostasis, as indicated by reduced K^+^ efflux and/or increased K^+^ influx in the roots?

## 2. Materials and Methods

**Figure 1:**
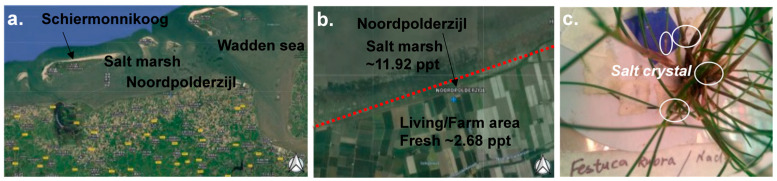
Locations of wild *Festuca rubra* populations in Dutch salt marshes and growth response of a commercial *F. rubra* cultivar under salt stress. (**a**) Habitat locations of wild *F. rubra* populations in the Wadden Sea (northern Netherlands) at Schiermonnikoog (53°29′24″ N, 6°13′30″ E) and (**b**) Noordpolderzijl (53°25′48″ N, 6°34′48″ E), where *F. rubra* populations have previously been observed and collected. The red dashed line is the dike between the salt marsh (above) and the inland agricultural area (below). The salinity level in these salt marshes is approximately 12 ppt (≈205 mM NaCl). (**c**) Growth response of the *F. rubra* ssp. *rubra* cv. Rafael at 100 mM NaCl grown in hydroponics. The white circles indicate salt crystals on the leaves. Previous studies confirmed that this cultivar is salt-sensitive [58,59,60].

Seeds of the commercial red fescue (*Festuca rubra* L. ssp. *rubra*) cultivar Rafael were obtained via DSV (Aurich, Germany). This cultivar was previously characterized as salt-sensitive based on growth measurements (Figure 1c; [59,60]).

To test the potential role of a fungal isolate on a commercial red fescue cultivar, we used a root-associated fungal isolate previously obtained from the roots of wild *F. rubra* growing in salt marshes along the Wadden Sea coast in the northern Netherlands (Figure 1a,b). This isolate, hereafter referred to as *Fusarium* sp. 1, was provisionally assigned to the genus *Fusarium* based on morphological characterization and preliminary taxonomic assessment (unpublished data, Section S1). As molecular identification was unavailable for the present study, this isolate is conservatively referred to as *Fusarium* sp. 1 throughout this study. The salinity tolerance of *Fusarium* sp. 1 was assessed across five NaCl concentrations ranging from 0 to 450 mM (Section S1).

For inoculum preparation, *Fusarium* sp. 1 was cultured in potato dextrose broth (24 g dextrose/L) supplemented with 50 µg/mL streptomycin. The culture was incubated at 27–29 °C for 46–50 h on a rotary shaker (140 rpm).

### 2.1. Conditions for Growth Experiments

#### 2.1.1. Seed Germination

Seeds of *Festuca rubra* ssp. *rubra* cv. Rafael, provided by DSV-Seeds (Aurich, Germany), were sown in plastic pots filled with tap water-saturated vermiculite. Pots were covered with transparent plastic foil and placed in a greenhouse (20/18 °C for day/night setpoint temperatures; a 14/10 h light/dark cycle) for six to seven days until germination. The photon fluence rate at plant height was at least 220 μmol m^−2^ s^−1^ (400–700 nm wavelength range), provided by MASTER SON-T PIA Plus GreenPower 600W E E40 lamps (Philips, Turnhout, Belgium).

#### 2.1.2. Seed Inoculation and Incubation for Growth Measurements

One milliliter of *Fusarium* sp.1 inoculum was added to the 25% Hoagland solution [61] (pH 5.9). The culture was incubated at 30 °C under continuous mixing for three days until use. The control solution consisted of the same Hoagland solution but supplemented with 1 mL of sterile demineralized water.

Seven days after seeds germinated, uniform seedlings were first dipped in the *Fusarium* sp. 1 inoculum and then transplanted into pots filled with soil and maintained under the same greenhouse conditions as during seed germination. An additional 0.5 mL of inoculum was added to each pot in the inoculation-treated group (*Fusarium*). Control seedlings were treated identically but dipped in the control solution lacking fungal inoculum (control). All plants were irrigated with a 25% Hoagland solution.

One week after inoculation, fungal hyphae within root tissues were checked using trypan blue staining (0.05% in lactic acid; modified from Johnson et al. [62]. Roots were cleared in 10% KOH for 10 min and then stained. Microscopic observations revealed fungal hyphae within the root cortical tissue of randomly selected inoculated plants, but no evidence of this was found in non-inoculated plants (Section S1), indicating successful root colonization. These observations were consistent with colonization by the inoculated fungal isolate. However, quantitative assessment of colonization intensity or fungal biomass was not performed.

#### 2.1.3. Salt Treatment and Growth Measurements

After confirming successful inoculation, plants were maintained in the greenhouse for an additional 14 days and watered daily with demi water. Then the salinity treatment was started: control plants were watered daily with 0.25 L of demi water and 0.5 L of a 25% Hoagland nutrient solution and salt treatment plants were watered daily with 0.25 L of demi water (to prevent the build-up of higher NaCl levels) and 0.5 L of a 25% Hoagland nutrient solution supplemented with 100 mM NaCl. This was repeated weekly for five weeks. At the end of the experiment, plants were removed from the pots and roots were gently rinsed with demineralized water and collected. Fresh and dry weights of shoots and roots were determined. Each treatment group included 10 plants (replicates). Growth experiments were conducted in soil to approximate realistic plant performance, whereas ion flux measurements were performed under hydroponic conditions to enable precise physiological assessment using the MIFE technique. The weights of the shoot and root were presented as the relative weight (Equation (1)):

(1) Relative weight (%)=[(W_treatment−W_control)/W_control]×100

where W_treatment is the biomass (shoot or root dry weight) measured under a given treatment, and W_control is the biomass (shoot or root dry weight) of plants grown under non-saline and non-inoculated conditions.

### 2.2. Preparation and Calibration of Microelectrodes for Ion Flux Measurements

K^+^-selective electrodes were prepared following the MIFE (Microelectrode Ion Flux Estimation) protocol [52,53,63]. Electrodes were calibrated using 250, 500 and 1000 µM KCl standard solutions. Only electrodes with a response slope of 50–59 mV per decade of concentration change and a correlation coefficient (R^2^) ≥ 0.999 were used. Detailed preparation procedures are described by Wang [60] and Wang et al. [58].

### 2.3. Plant Material Preparation for Ion Flux Measurements

Commercial *F. rubra* seeds of cv. Rafael (same batch as used in Section 2.1) were sown on vermiculite and 7-day-old seedlings were inoculated with *Fusarium* sp. 1, as described in Section 2.1.2. Inoculation success was verified one week later, as described in Section 2.1.2. After verification, plants were transferred to the hydroponic culture in a climate chamber (22/18 °C for day/night temperature; 40% relative humidity (RH); 14/10 h light/dark cycle; photon fluence rate: 100 ± 20 μmol·m^−2^·s^−1^ provided by Philips GreenPower LED production modules (deep red/white 120)). Inoculated (*Fusarium*) and non-inoculated (control) plants were placed in metal containers (30 plants per container, four containers per treatment) filled with 13 L of a 25% Hoagland nutrient solution. The nutrient solution was constantly aerated and refreshed twice a week. For EF-infected plants, 1 mL of *Fusarium* sp. 1 culture suspension was added to each container after transfer. Plants were incubated for 7 days before ion flux measurements.

#### 2.3.1. K^+^-Flux Scans Along Primary Roots

Seven days after incubation (Section 2.3 above), plants were pre-incubated at 50 mM NaCl for 1 h. Primary roots were then placed in a small Petri dish, fixed by a glass capillary attached to the dish base and immersed in a bath solution containing 0.1 mM CaCl_2_, 0.5 mM KCl and 0.550 mM MES (pH 6.0). K^+^-flux scans were performed along the distal 6000 µm of the root, with measurements taken at 250 or 500 μm intervals (from 0 to 6000 μm from the root tip). Each measurement was recorded for approximately 2 min per position. Based on the K^+^-flux profile of five replicate plants, a standard position at 400 μm distance from the root tip was selected for the subsequent experiments to determine the effect of both pre-treatments on Na^+^-induced root K^+^ fluxes (Section S3).

#### 2.3.2. NaCl-Induced Root K^+^ Fluxes

NaCl-induced K^+^ fluxes were measured in fresh primary roots of five control and EF-inoculated plants. Measurements were taken at 400 μm from the root tip in the same bath solution as described in Section 2.3.1. Baseline K^+^-flux was recorded for 15 min, followed by measurements for 25 min after 50 mM NaCl addition. Seminal roots without salt pre-treatment were also tested as a reference. Measurements were performed using five biological replicates.

#### 2.3.3. Effects of Long-Term Salinity Acclimation on NaCl-Induced Root K^+^ Fluxes

To evaluate the combined effects of salinity acclimation and *Fusarium* sp. 1 inoculation on NaCl-induced K^+^ fluxes, plants were subjected to long-term salinity pre-treatment one week after transfer to hydroponics. To minimize osmotic shock and allow physiological acclimation, NaCl was added gradually in 50 mM increments per day until final concentrations of 0, 50 or 100 mM were reached. Plants were subsequently maintained at their target salinity level before ion-flux measurements were performed. NaCl-induced K^+^ fluxes were measured in the roots of five individual plants using the procedures described in Section 2.3.2. The relative increase in K^+^ flux was calculated following Equation (2).

(2) Relative flux (%)=[(Flux_inoculated−Flux_control)/Flux_control]×100

where Flux_inoculated and Flux_control represent net K^+^ fluxes measured in inoculated and non-inoculated plants, respectively.

### 2.4. Statistical Analysis

Growth and K^+^-flux data were analyzed using Prism v8.0 (GraphPad Software, San Diego, CA, USA) and SPSS 28.0.1.0 (IBM, Armonk, NY, USA). Data are presented as mean ± SE. Differences between treatments were analyzed by two-way ANOVA followed by Tukey’s HSD test. Statistical significance was assessed at *p* < 0.05.

## 3. Results

### 3.1. Growth Responses of Commercial Festuca rubra to Salinity and Fusarium sp. 1 Inoculation

**Figure 2:**
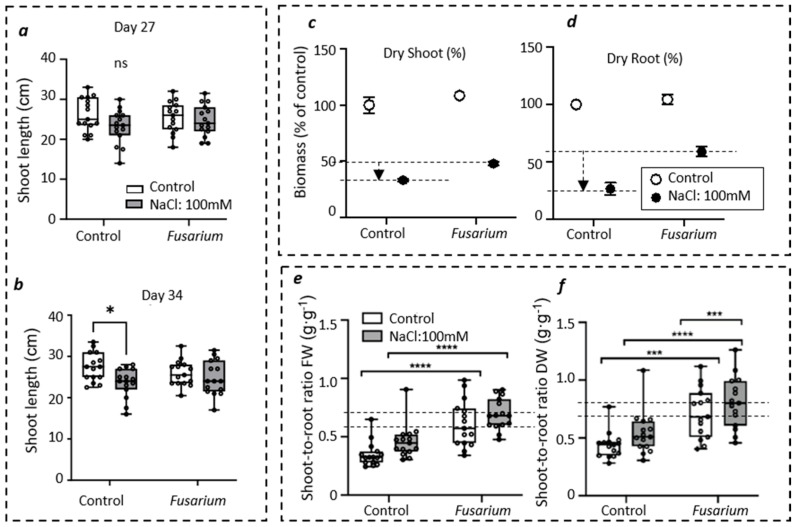
Effect of 100 mM NaCl and *Fusarium* sp. 1 inoculation on the shoot length of the commercial *Festuca rubra* ssp. *rubra* cv. Rafael after 27 (**a**) and 34 days (**b**); (**c**,**d**) shoot and root dry weights expressed relative to non-inoculated plants and non-saline control; (**e**,**f**) shoot-to-root ratio based on fresh weight (FW) and dry weight (DW). The downward black arrow indicates the biomass of plants inoculated compared to the corresponding non-inoculated control plants under salinity. Data represent the mean of 10 plants and were analyzed by two-way ANOVA (see Section S4; Tables S1–S6). *, *** and **** indicate significant differences at the *p* < 0.05; *p* < 0.001 and *p* < 0.0001 level, respectively. ns: not significant.

The effect of *Fusarium* sp. 1 inoculation on plant performance under salinity was evaluated by measuring shoot length and biomass of the commercial *F. rubra* cv. Rafael at 100 mM NaCl (Figure 2a,b, Section S2). Salt significantly reduced the shoot length of non-inoculated plants by 12.7% after 34 days. In contrast, inoculated plants did not show a significant decrease in shoot length compared to non-saline control plants.

The relative dry weights of shoots and roots (expressed as the percentage of the non-saline control) are shown in Figure 2c,d. Under saline conditions, plant inoculation with *Fusarium* sp. 1 partially alleviated salt-induced growth inhibition. The relative shoot weight of inoculated plants was approximately 15% higher than that of non-inoculated plants, whereas relative root dry weight increased by approximately 30% compared to non-inoculated plants at 100 mM NaCl (Figure 2c,d). Two-way ANOVA revealed significant effects of salinity and inoculation on shoot biomass (*p* < 0.05), whereas their interaction was not significant. For root biomass, however, both the effect of salinity and inoculation as well as their interaction were significant (*p* < 0.05; Section S4 Tables S3 and S4), indicating that inoculation mitigated salt-induced inhibition of root growth.

Salt treatment increased the shoot-to-root ratio, reflecting a shift in biomass allocation towards shoots (Figure 2e,f). *Fusarium* sp. 1 inoculation further increased the shoot-to-root ratio under salinity. The effect of inoculation and salinity on the shoot-to-root ratio was significant (*p* < 0.05, respectively; Section S4 Tables S5 and S6).

Overall, inoculation altered biomass allocation under salinity, resulting in a higher shoot-to-root ratio despite a relatively greater recovery of root biomass.

### 3.2. Root K^+^-Flux Responses to NaCl Stress and Fusarium sp.1 Inoculation

**Figure 3:**
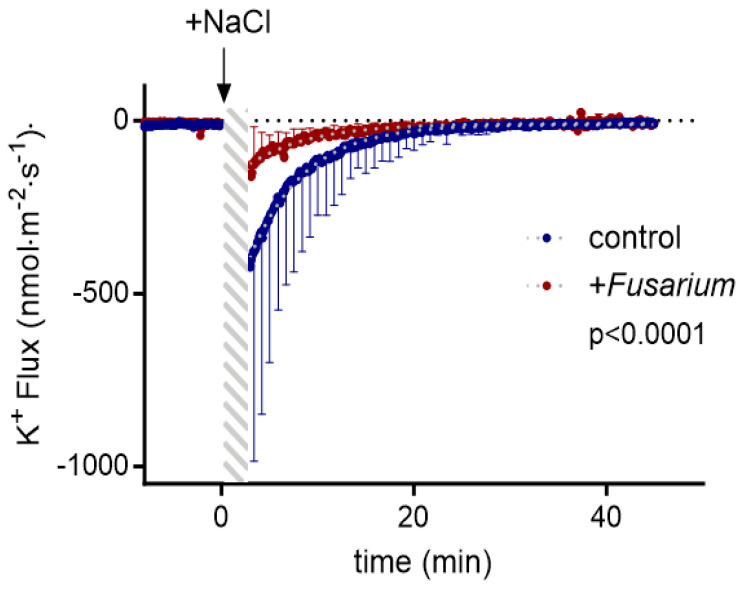
Na^+^-induced K^+^ fluxes in *Fusarium* sp. 1-inoculated (red) and non-inoculated (control; blue) primary roots of the commercial *Festuca rubra* ssp. *rubra* cv. Rafael. The K^+^ fluxes were measured at a distance of 400 μm from the root tip in the control bath solution (for details, see Materials and Methods Section 2.3.1) for about 5–10 min until the flux stabilized. When fluxes were stable, 50 mM NaCl was added to the bath solution and gently mixed, as indicated by the gray vertical bar. Positive values indicate a net K^+^ influx, while negative values indicate a net K^+^ efflux. Data represent the mean of five replicates (n = 5) with their standard error. Data were statistically analyzed by two-way ANOVA (see Section S4, Table S7 and online fit analysis Table S8). Parallel NaCl-induced K^+^ fluxes under control conditions were additionally measured in seminal roots of non-inoculated plants to exclude potential root-type-specific effects (Section S3: Figure S3b). Seminal roots displayed a comparable pattern to that of primary roots.

In non-inoculated plants, the addition of 50 mM NaCl induced a pronounced transient net K^+^ efflux in the root apex, reaching a peak of approximately 1000 nmol·m^−2^·s^−1^ shortly after NaCl application, followed by a gradual recovery toward the initial steady-state level within approximately 60 min (Figure 3).

In contrast, *Fusarium* sp. 1-inoculated plants exhibited a substantially reduced NaCl-induced K^+^ efflux (Figure 3). Only a small transient K^+^ efflux was observed, and fluxes recovered more rapidly towards steady-state levels compared to non-inoculated plants. While the overall temporal pattern of the response was similar, the magnitude of the flux was consistently lower in inoculated plants.

### 3.3. Effect of Fusarium sp. 1 Inoculation on Root K^+^ Flux Dynamics of Salt-Acclimated Plants

**Figure 4:**
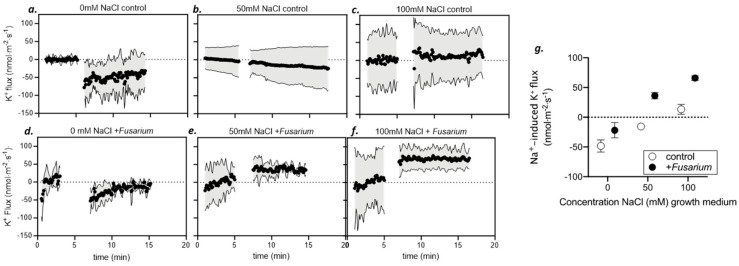
Actual traces showing the effect of the NaCl concentration in the growth medium and *Fusarium* sp. 1 inoculation on the Na^+^-induced K^+^ fluxes from the primary root of the commercial *Festuca rubra* ssp. *rubra* cv. Rafael. The upper and lower panels indicate *Fusarium* sp. 1-inoculated plants (+*Fusarium*) and non-inoculated plants (control), respectively. The gaps immediately after the 5 min time point indicate the period immediately following the addition of 50 mM NaCl. Data during this period are omitted because of transient electrical interference immediately following NaCl addition. The black line depicts the mean K^+^ flux of five replicates with the confidence interval (95%) in gray. The effect of *Fusarium* sp. 1 inoculation on Na^+^-induced K^+^ fluxes was summarized in Figure 4g as mean net K^+^ fluxes of five replicates (n = 5) with their standard error. Positive values indicate a net K^+^ influx, while negative values indicate a net efflux. The net K^+^ flux of inoculated plants was significantly different (*p* < 0.05, Tukey multiple comparison) from the control for all NaCl treatments. Data were analyzed by two-way ANOVA (see Section S4; Table S10).

To assess whether *Fusarium* sp. 1 inoculation affected root K^+^ flux in salt-acclimated plants, net K^+^ fluxes were measured in primary roots of *F. rubra*. Plants were grown under defined NaCl concentrations (0, 50 or 100 mM) prior to the flux measurements, after which a transient NaCl challenge was applied during the recording period (Figure 4).

In non-inoculated plants, increasing salinity levels were associated with progressively larger and more sustained net K^+^ effluxes (Figure 4a–c). Plants pre-grown without NaCl showed relatively small but unstable K^+^ fluxes, with a tendency towards net K^+^ efflux over time. In plants pre-exposed to 50 mM NaCl, a pronounced and sustained net K^+^ efflux was observed throughout the measurement period. This response was further amplified in plants grown at 100 mM NaCl, where roots exhibited the largest and most persistent K^+^ effluxes with increased temporal variability, indicating increased disruption of ion homeostasis.

In contrast, *Fusarium* sp. 1-inoculated plants exhibited markedly different K^+^ flux dynamics following long-term salt acclimation. Under non-saline pre-growth conditions, inoculated roots showed reduced K^+^ efflux and more stable flux patterns. In plants acclimated to 50 mM NaCl, K^+^ fluxes rapidly stabilized near zero and subsequently shifted towards net K^+^ influx following NaCl addition. Notably, in plants acclimated to 100 mM NaCl, *Fusarium* sp. 1-inoculated roots maintained stable K^+^ fluxes close to zero or shifted towards a net influx (uptake), indicating substantially reduced salt-induced K^+^ loss even under high salinity (Figure 4d–f).

Across all salinity levels, inoculation consistently shifted the net Na^+^-induced root K^+^ fluxes towards more positive values (Figure 4g). Both salinity and *Fusarium* sp. 1 inoculation had significant effects on Na^+^-induced K^+^ fluxes (*p* < 0.05). Inoculated plants acclimated at 50 and 100 mM NaCl increased root K^+^ influx by 333% and 397%, respectively, compared to non-inoculated plants (Section S4 Table S9, Equation (2)), whereas non-inoculated plants showed only minor increases or maintained near-zero net fluxes. This trend was consistently observed across all salinity treatments, indicating that *Fusarium* sp. 1 inoculation alters the direction and magnitude of Na^+^-induced K^+^ fluxes under both non-saline and saline growth conditions.

Together, these results show that long-term salinity acclimation was associated with progressively greater K^+^ efflux in non-inoculated *F. rubra*, whereas *Fusarium* sp. 1 inoculation maintained more stable root K^+^ fluxes and substantially reduced sustained K^+^ loss under saline conditions.

## 4. Discussion

In this study, we tested the hypothesis that salinity tolerance in plants can be enhanced through association with beneficial microorganisms. Consistent with previous reports that demonstrated the specific role of microbes in plant stress resistance [14,20,26,28,34,39,42,43], we showed that inoculation with a fungal isolate (i.e., *Fusarium* sp. 1) isolated from a salt-adapted ecotype of *F. rubra*, alleviated salinity-induced growth inhibition effects of salinity and reduced the NaCl-induced K^+^ loss of the salt-sensitive creeping red festuca cultivar Rafael. Under 100 mM NaCl, both shoot and root biomass of *Fusarium* sp. 1-inoculated plants were partially restored compared with non-inoculated plants, indicating a partial improvement in whole-plant performance under saline conditions. These findings demonstrate that a fungal isolate originally from a naturally salt-adapted wild red *festuca* population has the potential to enhance salinity tolerance in a commercial cultivar. Although inoculated plants exhibited reduced K^+^ loss under salt stress and improved growth performance, the present study establishes a correlation rather than direct causation. Therefore, maintenance of K^+^ homeostasis should be regarded as a likely contributing mechanism rather than a definitively proven primary mechanism underlying enhanced salt tolerance.

Salinity stress is known to disrupt Na^+^/K^+^ homeostasis primarily through Na^+^-induced K^+^ efflux from root cells, which compromises enzyme activity, membrane stability and overall plant performance [7,53,64,65]. Effective salinity tolerance, therefore, relies on the ability to retain K^+^ in the root tissues and to maintain a high cytosolic K^+^/Na^+^ ratio [48,50,54,55,56]. Our results showed that *Fusarium* sp. 1-inoculated plants exhibited reduced Na^+^-induced K^+^ efflux and accelerated recovery of root ion fluxes under salt stress. This improved K^+^ retention capacity might represent a key physiological mechanism underlying the improved salinity tolerance observed in inoculated plants. The beneficial effect of *Fusarium* sp. 1 was observed after both a short-term NaCl challenge and long-term salinity acclimation, combined with gradual stepwise salt application during long-term acclimation, suggesting that the observed responses were not merely transient effects associated with osmotic shock but reflected altered physiological adjustment to saline conditions. These findings are consistent with previous studies showing that fungal isolates from halophytic plants enhance salinity tolerance in crop species [43,44]. Murphy et al. [43], for example, demonstrated that endophytic fungi isolated from wild *Hordeum secalinum* improved growth and yield of cultivated barley under moderate salinity, although the beneficial effects diminished under more severe salt stress. Similarly, our results suggested that microbial resources associated with naturally salt-adapted grasses may improve crop performance under moderate salinity.

Maintenance of root K^+^/Na^+^ homeostasis under salinity generally involves several complementary processes, including restriction of Na^+^ entry, Na^+^ extrusion through plasma membrane Na^+^/H^+^ antiporters (SOS1), sequestration of Na^+^ into vacuoles via NHX transporters, selective Na^+^ retrieval by HKTs, and suppression of excessive K^+^ loss through outward-rectifying K^+^ channels [6,64,65]. These coordinated processes enable plants to maintain a high cytosolic K^+^/Na^+^ ratio, which is essential for enzyme activity, membrane stability and sustained growth under saline conditions. Although root K^+^ fluxes provide a sensitive physiological indicator of salinity tolerance, the corresponding transporters and antiporters were not determined in the present study. Therefore, the observed improvement in K^+^ retention might be interpreted as evidence of altered ion flux regulation rather than direct evidence of changes in whole-plant ion accumulation or K^+^/Na^+^ ratios. Because only ion fluxes were measured, changes in tissue K^+^ and Na^+^ concentrations remain undetermined. Future studies integrating both ion-flux and ion-content measurements would provide a more comprehensive understanding of the mechanisms involved.

Reduced K^+^ efflux in inoculated plants might involve the modulation of outward-rectifying K^+^ channels, as previously suggested for salt-tolerant genotypes [64,66,67]. Similar microbe-mediated regulation of ion homeostasis has been reported in other systems, including upregulation of Na^+^/K^+^ exchangers and improved Na^+^ exclusion. In the present study, the commercial *F. rubra* cultivar initially showed limited ability to maintain K^+^ homeostasis under salt stress, whereas inoculation with the fungal isolate improved K^+^ retention dynamics. Although the precise molecular mechanisms remain to be elucidated, the reduced K^+^ efflux observed in inoculated plants may reflect improved regulation of these ion transport processes. Beneficial fungal endophytes have previously been reported to enhance salt tolerance by reducing Na^+^ accumulation, increasing K^+^ retention, and regulating genes associated with ion transport and homeostasis [16,17,20,26]. Our findings are consistent with these observations, although direct measurements of transporter activity or gene expression were beyond the scope of the present study. Future transcriptomic or molecular studies focusing on transporters such as SOS1, NHX and HKT will be valuable to determine whether similar regulatory pathways contribute to the enhanced K^+^ retention observed in *Fusarium* sp. 1-inoculated plants.

Improved K^+^ retention is closely linked to enhanced root function, nutrient transport, and biomass allocation under salt stress [21,49,50,68]. In the present study, inoculated plants exhibited a higher shoot-to-root biomass ratio under both control and saline conditions, indicating a relative shift in biomass allocation toward the shoots. Importantly, this occurred alongside an increase in total plant biomass, suggesting improved overall plant performance. Such responses may result from enhanced root integrity, altered hormonal regulation, or improved physiological functioning, all of which have been reported in plant–microbe interactions [9,13,37,46]. As the plants were grown under identical nutrient conditions, differences in ion fluxes were unlikely solely due to nutrient availability. Endophytic fungi are known to produce phytohormones and other bioactive metabolites that influence root development, stress responses, and ion homeostasis [12,15,16,22,35,36,69,70]. Rather than inducing entirely novel stress-tolerance mechanisms, these microbial associates may enhance existing plant physiological processes, including water relations, antioxidant capacity, and ion regulation [17,18,19,24,44,46]. Although the molecular basis of the reduced K^+^ efflux observed in inoculated plants remains unclear, it is conceivable that *Fusarium* sp. 1 influences the activity or regulation of membrane transport systems involved in K^+^ retention under salt stress through the production of signaling molecules or metabolites. While phytohormone production, antioxidant responses, and fungal metabolites were not assessed in the present study, these represent plausible complementary mechanisms contributing to the observed improvements in growth and homeostasis. Future studies integrating metabolomic, transcriptomic, and ion transport analyses will be necessary to identify the fungal factors involved and clarify the signaling pathways underpinning this plant–endophyte interaction.

Beyond potassium, microbial associates have been reported to enhance the acquisition and utilization of other essential nutrients, including nitrogen and phosphorus, thereby further supporting plant performance under abiotic stress [8,27]. Whether similar nutrient-related mechanisms contribute to the beneficial effects of *Fusarium* sp. 1 in *F. rubra* remains an important topic for future investigation.

Although many *Fusarium* species are recognized as important plant pathogens, non-pathogenic isolates capable of establishing symptomless root colonization are increasingly being recognized as beneficial microorganisms with potential applications in plant growth promotion and biological control [28,45,71]. Several *Fusarium* species have been reported to function as symptomless endophytes that do not impair host performance and may even enhance plant growth under field conditions [72]. For example, inoculation with *Fusarium verticillioides* improved soybean germination and growth under salinity stress [45], while more recent studies have shown that *Fusarium* isolates from native plants can promote plant growth and induce systemic resistance to pathogens [28]. Extending these findings, our study demonstrates that a *Fusarium* isolate originating from a naturally saline coastal salt-marsh ecosystem can enhance salinity tolerance in a commercial *Festuca rubra* cultivar, an effect associated with improved root K^+^ homeostasis. To our knowledge, reports linking root-associated *Fusarium* isolates from salt-marsh grasses to enhanced K^+^ retention under salinity stress remain scarce. However, an important limitation of the present study is that only a single endophytic isolate was investigated. Given the substantial taxonomic and functional diversity among endophytic fungi, and even among closely related *Fusarium* strains, the beneficial effects observed here should not be considered representative of the genus *Fusarium* or endophytic fungi more broadly. Future studies comparing multiple isolates will be necessary to determine the extent to which these responses are strain-specific and to evaluate their broader applicability.

Nevertheless, *Fusarium* occupies diverse ecological niches, including endophytes, saprotrophs, and important plant pathogens, with some species capable of producing mycotoxins. Although the isolate used in this study was recovered from symptom-free root tissues and did not induce visible disease symptoms during the experimental period, its pathogenic potential and metabolite profile were not evaluated. Therefore, the present findings should be carefully interpreted as evidence supporting the direct agricultural application of *Fusarium* isolates. Rather, this study provides proof-of-concept evidence that a root-associated fungal isolate obtained from a naturally salt-adapted plant population can enhance salinity tolerance in a commercial grass cultivar. Future studies should include multilocus molecular identification (e.g., ITS, TEF1-α, and RPB2), pathogenicity testing, mycotoxin screening and environmental risk assessment before agricultural application can be considered. Furthermore, fungal biomass and colonization intensity were not quantified for quantitative colonization analyses (microscopy or qPCR). Consequently, relationships between the extent of root colonization and the magnitude of physiological responses could be assessed. Antioxidant responses were not evaluated in the present study, and therefore, further studies integrating ion-flux measurements with analyses of antioxidant metabolism would help elucidate the mechanisms underlying the observed salinity tolerance. In addition, the altered K^+^ flux patterns observed in inoculated plants may reflect changes in the activity of ion transport systems involved in K^+^ homeostasis. However, because transporter expression and channel activity were not directly assessed, the involvement of specific transporters or channels remains speculative and requires further investigation. Another limitation of the present study is that we focused exclusively on root colonization and did not investigate whether inoculation influenced microbial communities and their biomass in aboveground tissues. Increasing evidence indicates that root-associated microorganisms can affect the assembly and function of the phyllosphere microbiome, and that root and leaf microbiota may share overlapping functional traits [73,74,75,76]. Whether inoculation with *Fusarium* sp. 1 alters the aboveground endophytic microbiome and whether such changes contribute to improved salinity tolerance remain open questions requiring further investigation.

The experiments presented in this study were conducted under controlled hydroponic conditions using a single *Festuca rubra* cultivar and a single fungal isolate and were not repeated across multiple environments or growing seasons. Consequently, the generality and long-term stability of the observed effects remain to be established. Future studies should include detailed taxonomic characterization of this *Fusarium* isolate, investigation of its ecological role (e.g., mutualistic, commensal, latent pathogenic, or as a component of the phyllosphere microbiome), and evaluation across diverse plant genotypes, environments, and field or semi-natural conditions. Such studies will be essential to determine the persistence, consistency, and broader applicability of this endophyte as a potential tool for enhancing plant salt tolerance.

## 5. Conclusions

Salinity is a major abiotic stress that substantially limits global crop productivity, underscoring the urgent need for more salt-tolerant varieties. In this study, we demonstrated that inoculation with a fungal isolate (*Fusarium* sp. 1), obtained from wild *F. rubra* naturally growing in a Dutch coastal salt marsh, partially alleviated salt-induced growth inhibition and K^+^ loss in a salt-sensitive commercial creeping red fescue cultivar under the experimental conditions used in this study.

Our results highlight that wild plant populations adapted to extreme environments represent valuable reservoirs of beneficial microbiome resources. Exploring such naturally occurring plant–microbe interactions may therefore complement conventional breeding approaches for improving salinity resilience in grasses and other crops. Because this study examined a single fungal isolate under controlled experimental conditions, further investigations are required before practical applications can be considered. Future studies should include species-level identification of the fungal isolate, quantitative assessment of root colonization, evaluation of pathogenicity and biosafety, elucidation of the underlying molecular mechanisms, and validation across multiple plant genotypes and species under greenhouse and field conditions.

## Data Availability

The raw data supporting the conclusions of this article will be made available by the authors on request.

## Supplementary Materials

The following supporting information can be downloaded at https://www.mdpi.com/article/10.3390/microorganisms14071598/s1. Section S1: The morphology of the salt marsh fungal isolate *Fusarium* sp. 1 exposed to artificial seawater; Section S2: Plant growth under salt and *Fusarium* sp. 1 inoculation; Section S3: K^+^-flux profile along the root and a versification on primary and seminal root; Section S4: Statistical analyses. Figure S1: Morphology of the salt-marsh fungal isolate *Fusarium* sp. 1. exposed to artificial seawater;. Figure S2: Commercial *Festuca rubra* ssp. *rubra* cv. Rafael shoot length development at 0 and 100 mM NaCl of non-inoculated control plants (a) and *Fusarium* sp. 1-inoculated plants (b); Figure S3: Transient net K^+^ fluxes along the primary root of *Festuca rubra* ssp. *rubra* cv. Rafael (a) and NaCl-induced (50 mM) K^+^ fluxes in *Fusarium* sp. 1 inoculated (primary) and non-inoculated primary and seminal roots of *F. rubra* cv. Rafael (b) Table S1: Two-way ANOVA for shoot length as affected by *Fusarium* sp. 1 inoculation and salinity (Figure 2a and Figure S2. day 27); Table S2: Two-way ANOVA for shoot length as affected by *Fusarium* sp. 1 inoculation and salinity (Figure 2b and Figure S2 day 34); Table S3: Two-way ANOVA for shoot dry biomass as affected by *Fusarium* sp. 1 inoculation and salinity (% of control, Figure 2c shoot); Table S4: Two-way ANOVA table for the root dry biomass as affected by *Fusarium* sp. 1 inoculation and salinity (% of control, Figure 2d root); Table S5: Two-way ANOVA for the effect of salinity (100 mM NaCl) and *Fusarium* sp. 1 inoculation on the shoot-to-root ratio expressed on a FM basis (Figure 2e); Table S6: Two-way ANOVA for the effect of salinity (100 mM NaCl) and *Fusarium* sp. 1 inoculation on the shoot-to-root ratio expressed on a DM basis (Figure 2f); Table S7: Two-way Anova for Na^+^-induced K^+^ fluxes in *Fusarium* sp. 1 inoculated (primary) and non-inoculated (primary & seminal) roots of *Festuca rubra* ssp. *rubra*. (Figure 3); Table S8: Online fit analysis for NaCl-induced K^+^ fluxes in *Fusarium* sp. 1 inoculated (primary) and non-inoculated (primary & seminal) roots (Figure 3); Table S9: Mean NaCl-induced K+ flux values and the *Fusarium* sp. 1 inoculation-induced K+ flux increase calculated as % of the non-inoculated control *Festuca rubra* ssp. rubra (Figure 4); Table S10: Two-way ANOVA for the effect of NaCl concentration in the nutrient solution and *Fusarium* sp. 1 inoculation on the NaCl-induced K^+^ fluxes of primary roots (Figure 4g).

## Abbreviations

SM	Salt marsh
RH	Relative humidity
ssp.	Subspecies
sp.	Species
HKTs	High-affinity K^+^ transporters
NHXs	(Na^+^/H^+^ antiporters)
cv.	Cultivar
ANOVA	Analysis of variance
SS	Sum of squares
DF	Degrees of freedom
MS	Mean square (SS/DF)
F (DFn, DFd)	F-statistic (degrees of freedom of numerator and denominator, resp.)
*p* value	Probability value indicating statistical significance
