# Supplementary material for: A Fusarium Isolate from a Salt Marsh Improves the Salinity Tolerance of a Commercial Cultivar of Festuca rubra via Enhanced Root K+ Homeostasis
Source: Microorganisms. 2026 Jul 22;14(7):1598. doi: 10.3390/microorganisms14071598 (PMC13413935; doi:10.3390/microorganisms14071598)
Supplement: Supplementary file 1 [file microorganisms-14-01598-s001.zip › microorganisms-4377740-supplementary.pdf]

Supplementary

A *Fusarium* Isolate from a Salt Marsh Improves the Salinity Tolerance of a Commercial Cultivar of *Festuca rubra* via Enhanced Root K^+ Homeostasis

Section S1. *The morphology of the salt-marsh fungal isolate* Fusarium *sp. 1. exposed to artificial seawater.*

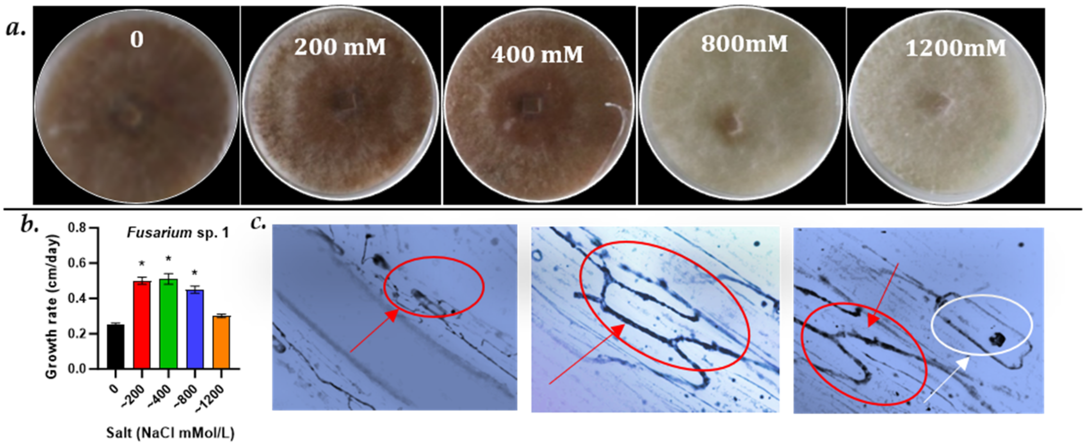

Figure S1. Morphology of the salt-marsh fungal isolate *Fusarium* sp. 1. exposed to artificial seawater (**a**). The root-colonizing fungal isolate was grown on PDA-plates supplemented with either 0, 0.5, 1, 2 and 3 times the concentration of salts in seawater (lab made artificial seawater contains 400 mM NaCl, 5 mM Na_2SO_4 and 5 mM $MgCl_2$). The salinity-tolerant growth of the *Fusarium* fungal isolate indicated a significant difference from the control at 200, 400, 800 and 1200 mM NaCl (**b**, data represent the mean of 4 measurements ±SEM). The plant-fungus symbiont structures were photographed at magnifications of 100, 200 and 400 times (**c**). The threadlike structures (the red circles) are intercellular hyphae and the spherical structures (the white circle) are structures used by the fungal isolate to store nutrients and other compounds.

Section S2. *Plant growth under salt and* Fusarium *sp. 1 inoculation.*

Shoot length measurements young seedlings provided independent morphological support for the biomass responses (Figure S2a, b). In non-inoculated plants, shoot length decreased significantly at 100 mM NaCl compared with control conditions, confirming the salt-sensitive nature of the commercial *Festuca rubra* ssp. *rubra* cv. Rafael (Figure S2a). Under salinity stress (100 mM NaCl), *Fusarium* sp. 1 inoculated plants exhibited reduced shoot growth inhibition, resulting in an almost similar shoot length development relative to non-inoculated plants (Figure S2b). Together, these results demonstrate that *Fusarium* sp. 1 inoculation improves growth performance and biomass allocation of *F. rubra* under saline conditions.

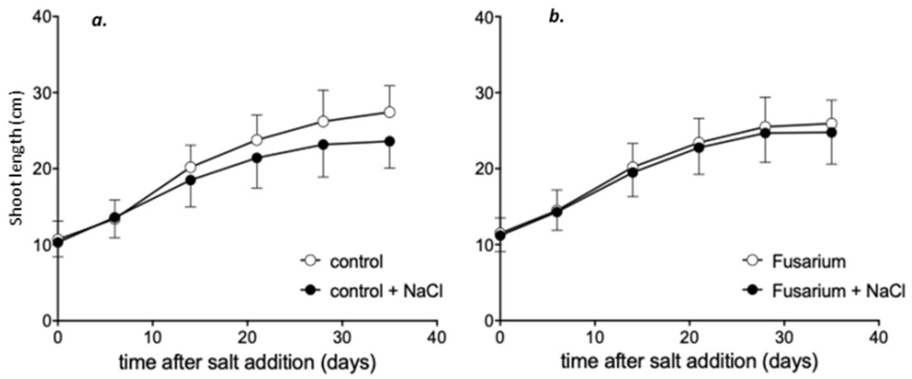

Figure *S***2.** Commercial *Festuca rubra* ssp. *rubra* cv. Rafael shoot length development at 0 and 100 mM NaCl of non-inoculated control plants (**a**) and *Fusarium* sp. 1-inoculated plants (**b**). Data represent the mean of 10 seedlings ±SD.

Section S3. *K⁺-flux profile along the primary root and a comparison in NaCl-induced K⁺ fluxes in primary and seminal roots.*

F. rubra ssp. *rubra* cv. Rafael roots exhibited a NaCl-induced (50 mM) K^+ efflux along the primary root (Figure S3a). The highest efflux was found around 200 μm from the root tip and the net effluxes decreased along the root till about 1000 μm. From 1000 μm onwards the efflux remains small. Based on the K^+ flux profile a point at 400 μm distance from the root tip was selected for the subsequent experiments on the effect of pre-treatment of the plants on Na^+-induced root K^+ fluxes. This location is characterized as not close to the maximum efflux but still in the region that is dominated by the fluxes that are associated with the zone just behind the root tip.

As the difference between primary and seminal roots in grasses like *F. rubra* is sometimes not obvious and mistakes can easily be made, we wanted to make sure that both root types do respond in a similar manner. It is obvious that the difference in the response to added NaCl (50 mM) between the two root types, if any, is only a quantitative one, as seminal roots seem to exhibit a somewhat larger Na^+-induced K^+ efflux (Figure S3b). In plants that were inoculated with *Fusarium* sp. 1 the addition of NaCl did only induce a minimal efflux of K^+. It can be observed that the smaller induced K^+ flux in roots of infected plants did follow the same kinetic decay to the initial steady state value, indicating a similar regulatory mechanism of the fluxes, albeit that these fluxes are at a much-reduced scale.

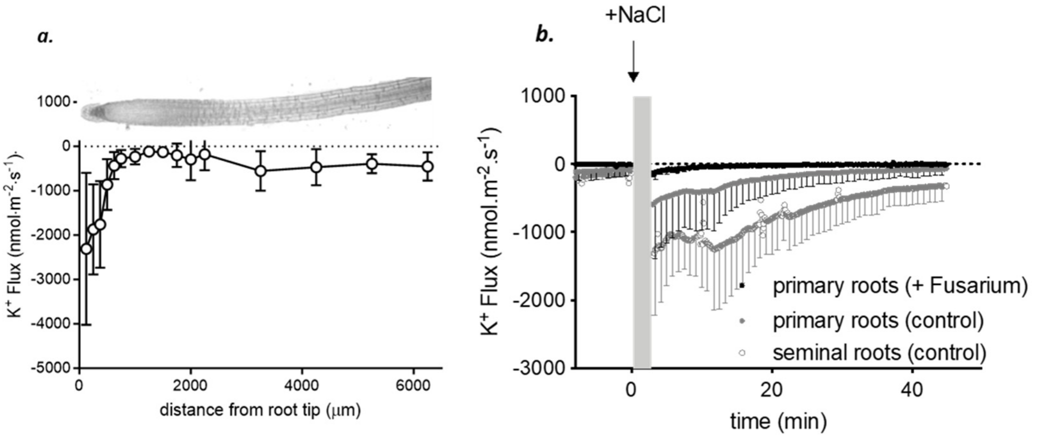

Figure S3: Transient net K^+ fluxes along the primary root of *Festuca rubra* ssp. *rubra* cv. Rafael (**a**) and NaCl-induced (50 mM) K^+ fluxes in *Fusarium* sp. 1 inoculated (primary) and non-inoculated primary and seminal roots of *F. rubra* cv. Rafael (**b**). Presented are the mean fluxes ±SD of three (**a**) and five roots (**b**).

Section S4. *Statistical analyses*

Table S1. Two-way ANOVA for shoot length as affected by *Fusarium* sp. 1 inoculation and salinity (Fig 2a, Figure S2. day 27).

Source of Variation	% of total	P value	P value summary	Significant?	
**Interaction**	1.879	0.2903	ns	No	
Time/Dates	0.2616	0.6919	ns	No	
**Treatment**	5.535	0.0722	ns	No	
**ANOVA table**	**SS (Type III)**	**DF**	**MS**	**F (DFn, DFd)**	**P value**
**Interaction**	18.70	1	18.70	F (1, 56) = 1.140	P=0.2903
Time/Dates	2.604	1	2.604	F (1, 56) = 0.1587	P=0.6919
Treatment	55.10	1	55.10	F (1, 56) = 3.357	P=0.0722
**Residual**	919.1	56	16.41		

ANOVA: Analysis of variance; SS: Sum of squares; DF: Degrees of freedom; MS: Mean square (SS/DF); F (DFn, DFd): F-statistic, where DFn and DFd denote the degrees of freedom of the numerator and denominator, respectively; P value: Probability value indicating statistical significance.

Table S2. Two-way ANOVA for shoot length as affected by *Fusarium* sp. 1 inoculation and salinity (Fig 2b, Figure S2 day 34).

Source of Variation	% of total	P value	P value summary	Significant?	
**Interaction**	0.7326	0.8041	ns	No	
Time/Dates	73.81	<0.0001	****	Yes	
**Treatment**	1.331	0.0004	***	Yes	
**ANOVA table**	**SS (Type III)**	**DF**	**MS**	**F (DFn, DFd)**	**P value**
**Interaction**	106.7	15	7.114	F (15, 336) = 0.6802	P=0.8041
Time/Dates	10752	5	2150	F (5, 336) = 205.6	P<0.0001
Treatment	193.9	3	64.62	F (3, 336) = 6.179	P=0.0004
**Residual**	3514	336	10.46		

ANOVA: Analysis of variance; SS: Sum of squares; DF: Degrees of freedom; MS: Mean square (SS/DF); F (DFn, DFd): F-statistic, where DFn and DFd denote the degrees of freedom of the numerator and denominator, respectively; P value: Probability value indicating statistical significance.

Table S3. Two-way ANOVA for shoot dry biomass as affected by *Fusarium* sp. 1 inoculation and salinity (% of control, Figure 2c shoot).

Source of Variation	% of total variation	P value	P value summary	Significant	
**Interaction**	0.2253	0.2049	ns	No	
Fusarium	3.180	0.0008	***	Yes	
Salinity	95.65	<0.0001	****	Yes	
**ANOVA table**	**SS**	**DF**	**MS**	**F (DFn, DFd)**	**P value**
**Interaction**	28.64	1	28.64	F (1, 8) = 1.904	P=0.2049
Fusarium	404.4	1	404.4	F (1, 8) = 26.88	P=0.0008
Salinity	12162	1	12162	F (1, 8) = 808.5	P<0.0001
**Residual**	120.3	8	15.04		

ANOVA: Analysis of variance; SS: Sum of squares; DF: Degrees of freedom; MS: Mean square (SS/DF); F (DFn, DFd): F-statistic, where DFn and DFd denote the degrees of freedom of the numerator and denominator, respectively; P value: Probability value indicating statistical significance.

Table S4. Two-way ANOVA table for the root dry biomass as affected by *Fusarium* sp. 1 inoculation and salinity (% of control, Figure 2d root).

Source of Variation	% of total variation	P value	P value summary	Significant?	
**Interaction**	4.735	0.0003	***	Yes	
Fusarium	8.333	<0.0001	****	Yes	
Salinity	85.90	<0.0001	****	Yes	
**ANOVA table**	**SS**	**DF**	**MS**	**F (DFn, DFd)**	**P value**
**Interaction**	587.2	1	587.2	F (1, 8) = 36.67	P=0.0003
Fusarium	1033	1	1033	F (1, 8) = 64.54	P<0.0001
Salinity	10653	1	10653	F (1, 8) = 665.3	P<0.0001
**Residual**	128.1	8	16.01		

ANOVA: Analysis of variance; SS: Sum of squares; DF: Degrees of freedom; MS: Mean square (SS/DF); F (DFn, DFd): F-statistic, where DFn and DFd denote the degrees of freedom of the numerator and denominator, respectively; P value: Probability value indicating statistical significance.

Table S5. Two-way ANOVA for the effect of salinity (100 mM NaCl) and *Fusarium* sp. 1 inoculation on the shoot-to-root ratio expressed on a FM basis (Figure 2e).

**Source of Variation**	**% of total variation**	**P value**	**P value summary**	**Significant?**	
**Interaction**	0,1347	0,7104	ns	No	
Fusarium	39,71	<0,0001	****	Yes	
Salinity	6,007	0,0157	*	Yes	
**ANOVA table**	**SS**	**DF**	**MS**	**F (DFn, DFd)**	**P value**
**Interaction**	0,003132	1	0,003132	F (1, 56) = 0,1393	P=0,7104
Fusarium	0,9235	1	0,9235	F (1, 56) = 41,07	P<0,0001
Salinity	0,1397	1	0,1397	F (1, 56) = 6,213	P=0,0157
**Residual**	1,259	56	0,02248		

ANOVA: Analysis of variance; SS: Sum of squares; DF: Degrees of freedom; MS: Mean square (SS/DF); F (DFn, DFd): F-statistic, where DFn and DFd denote the degrees of freedom of the numerator and denominator, respectively; P value: Probability value indicating statistical significance.

Table S6. Two-way ANOVA for the effect of salinity (100 mM NaCl) and *Fusarium* sp. 1 inoculation on the shoot-to-root ratio expressed on a DM basis (Figure 2f).

**Source of Variation**	**% of total variation**	**P value**	**P value summary**	**Significant?**	
**Interaction**	44.22	0.0085	**	Yes	
Fusarium	22.58	0.0384	*	Yes	
Salinity	3.698	0.3459	ns	No	
**ANOVA table**	**SS**	**DF**	**MS**	**F (DFn, DFd)**	**P value**
**Interaction**	0.02911	1	0.02911	F (1, 8) = 11.99	P=0.0085
Fusarium	0.01486	1	0.01486	F (1, 8) = 6.124	P=0.0384
Salinity	0.002434	1	0.002434	F (1, 8) = 1.003	P=0.3459
**Residual**	0.01942	8	0.002427		

ANOVA: Analysis of variance; SS: Sum of squares ; DF: Degrees of freedom; MS: Mean square (SS/DF); F (DFn, DFd): F-statistic, where DFn and DFd denote the degrees of freedom of the numerator and denominator, respectively; P value: Probability value indicating statistical significance.

Table S7. Two-way Anova for Na^+-induced K^+ fluxes in *Fusarium* sp. 1 inoculated (primary) and non-inoculated (primary & seminal) roots of *Festuca rubra* ssp. *rubra*. (Figure 3)

**Source of Variation**	**% of total variation**	**P value**	**P value summary**	**Significant?**
time	73.81	<0,0001	****	Yes
Fusarium+salinity	6.217	<0,0001	****	Yes

**ANOVA table**	**DF**	**MS**	**F (DFn, DFd)**	**P value**
time	623	5604	F (623, 623) = 3.696	P<0,0001
Fusarium+salinity	1	294063	F (1, 623) =193.9	P<0,0001
**Residual**	623	1516		

ANOVA: Analysis of variance; SS: Sum of squares; DF: Degrees of freedom; MS: Mean square (SS/DF); F (DFn, DFd): F-statistic, where DFn and DFd denote the degrees of freedom of the numerator and denominator, respectively; P value: Probability value indicating statistical significance.

Table S8. Online fit analysis for NaCl-induced K^+ fluxes in *Fusarium* sp. 1 inoculated (primary) and non-inoculated (primary & seminal) roots (Figure 3).

**Comparison of fits**	
Null hypothesis	2 parameters same for all data sets
Alternative hypothesis	2 parameters different for each data set
P value	< 0,0001
Conclusion (alpha = 0.05)	Reject null hypothesis
Preferred model	2 parameters different for each data set
F (DFn, DFd)	12536 (2,1004)

Table S9. Mean NaCl-induced K^+ flux values and the *Fusarium* sp. 1 inoculation-induced K+ flux increase calculated as % of the non-inoculated control *Festuca rubra* ssp. *rubra* (Figure 4).

	**Mean flux**			**Relative flux (%)**		
NaCl（mM)	**0**	**50**	**100**	**0**	**50**	**100**
Control	-48.3331	-15.534	13.24988			
Inoculated	-21.8416	36.2125	65.85583	-54.81	-333.118	397.030

Relative flux (%) = [(Flux_inoculated − Flux_control) / Flux_control] × 100 (Eq. 2), where Flux_inoculated and Flux_control represent net K^+ fluxes measured in inoculated and non-inoculated plants, respectively.

Table S10. Two-way ANOVA for the effect of NaCl concentration in the nutrient solution and *Fusarium* sp. 1 inoculation on the NaCl-induced K^+ fluxes of primary roots (Figure 4g)

**Source of Variation**	**% of total variation**	**P value**	**P value summary**	**Significant?**
**Interaction**	2,325	<0,0001	****	Yes
Fusarium	61,13	<0,0001	****	Yes
Salinity	29,91	<0,0001	****	Yes

**ANOVA table**	**SS (Type III)**	**DF**	**MS**	**F (DFn, DFd)**	**P value**
**Interaction**	19818	2	9909	F (2, 538) = 149,5	P<0,0001
Fusarium	521100	2	260550	F (2, 538) = 3931	P<0,0001
Salinity	254996	1	254996	F (1, 538) = 3847	P<0,0001
**Residual**	35664	538	66,29		

ANOVA: Analysis of variance; SS: Sum of squares; DF: Degrees of freedom; MS: Mean square (SS/DF); F (DFn, DFd): F-statistic, where DFn and DFd denote the degrees of freedom of the numerator and denominator, respectively; P value: Probability value indicating statistical significance.
